# Roles of PPARγ/NF-κB Signaling Pathway in the Pathogenesis of Intrahepatic Cholestasis of Pregnancy

**DOI:** 10.1371/journal.pone.0087343

**Published:** 2014-01-29

**Authors:** Yan Zhang, Lingqing Hu, Yan Cui, Zhigang Qi, Xiaoping Huang, Liyi Cai, Ting Zhang, Yongxiang Yin, Zhiyi Lu, Jingying Xiang

**Affiliations:** 1 Department of Gynecology and Obstetrics, Wuxi Maternal and Child Health Hospital, the Affiliated Hospital of Nanjing Medical University, Wuxi, Jiangsu, China; 2 Department of Pharmacology, College of Pharmaceutical Sciences, Soochow University, Suzhou, Jiangsu, China; 3 Department of Pharmaceutical, Wuxi People’s Hospital, Wuxi, Jiangsu, China; 4 Department of Obstetrics and Gynecology, Wuxi Hospital of Traditional Chinese Medicine, Wuxi, Jiangsu, China; Van Andel Institute, United States of America

## Abstract

**Background:**

Intrahepatic cholestasis of pregnancy (ICP) is the most prevalent pregnancy specific liver disease. However, the pathogenesis and etiology of ICP is poorly understood.

**Aim:**

To assess the expression of peroxisome proliferator-activated receptorγ (PPARγ) and nuclear factor kappa B (NF-κB) in placenta and HTR-8/SVneo cell, and evaluate the serum levels of cytokines, bile acids, hepatic function and lipids in control and ICP patients and the fetal outcome, in order to explore the role of PPARγ/NF-κB signaling pathway in the possible mechanism of ICP.

**Methods:**

Clinical data of the pregnant women were collected and serum levels of cytokines, bile acids, hepatic function and lipids were measured. Expressions of PPARγ and NF-κB in placenta and HTR-8/SVneo cell were determined. The new-born information was collected to demonstrate the relationship between PPARγ/NF-κB signaling pathway and ICP.

**Results:**

The serum levels of bile acids, hepatic function, triglycerides (TG), total cholesterol (TC), IL-6, IL-12 and TNF-α in ICP group were significantly increased (P<0.01), and serum level of IL-4 was significantly decreased (P<0.01). PPARγ and NF-κB staining were found in the membrane and cytoplasm of placental trophoblast cell. The expression of PPARγ and NF-κB were significantly higher in ICP group and taurocholate acid (TCA) treated HTR-8/SVneo cell (P<0.01). The new-born information in severe ICP group were significantly different as compared to that in control group (P<0.05), and part of information in mild ICP group were also difference to that in control group (P<0.05).

**Conclusions:**

The higher expressions of PPARγ and NF-κB in ICP placenta and TCA treated HTR-8/SVneo cell, together with the abnormal serum levels of cytokines, might induced by the imbalance of inflammatory and immune reaction, and then disturb placental bile acid and serum lipids transportation, finally result in fatal cholestasis which probably be one of the mechanism of ICP.

## Introduction

Intrahepatic cholestasis of pregnancy (ICP) is a pregnancy specific disease and has a prevalence that varies by geography and ethnicity [1], [2]. ICP is characterized by otherwise unexplained pruritus with elevated bile acids and/or transaminases in late second and third trimester of pregnancy which fully resolve after delivery, and is associated with an increased risk of foetal distress, preterm delivery and sudden intrauterine foetal death [3]–[5]. The occurrence rate of ICP varies worldwide ranging from 0.1% to 15% [6]. In China, ICP is considered to be common, with an incidence of 0.8%–12.0% [7]. Currently, the treatments to ICP only alleviate the mother’s itching, which cannot relieve the harm to the fetus [8]. Therefore, study the molecular mechanisms of pathogenesis in the ICP development process seems very important.

The pathogenesis of ICP has been associated with reproductive hormones, immune and environmental factors, bile acids and the genetic variation of bile acid homeostasis related genes [9]–[11]. However, the etiology of ICP is still poorly understood [12]. Recent studies have focused on the potential role of certain imbalance of immune reaction in the etiology of ICP [13], [14]. Some investigations found imbalance of immune reaction was present in maternal peripheral blood and placenta tissue, and concluded the missing balance of immunization was one of the reasons to induce ICP.

The immune system plays an important role in pregnancy, both in normal and pathologic states [15]. The immune responsiveness of women is altered during pregnancy in order to retain protective properties against disease and at the same time to allow tolerance of the fetus. The semi-allogeneic fetus is an immunological paradox in that its development seems to circumvent the rejection of the maternal immune system in successful human pregnancy. Cytokines, natural killer cells and T cells, are thought to be important mediators in the communication between the mother and the developing fetus [16]. T cell immune responses occur throughout pregnancy. T cells can differentiate into effectors whose cytokine profile is limited to type 1 (Th1) or type 2 (Th2). It is accepted that human pregnancy is associated with a shift away from Th1 type and a bias toward Th2-type immune responses, which helps ameliorate potentially lethal Th1 and cytotoxic T cell responses,while ICP is consistent with suppressed Th2-type cytokines [17], [18]. The molecular mechanisms that regulate this shift are as yet unknown.

The nuclear factor kappa B (NF-κB) family of transcription factors is a critical regulator of immune and inflammatory processes [19]. It can be activated by porinflammatory cytokines and endotoxins and then translocate to the nucleus to promote transcription of inflammatory mediators, regulates inducible gene expression under both physiological and pathological conditions. NF-κB plays a key regulatory role in controlling Th1 and Th2 immune responses [19], [20].

Peroxisome proliferator-activated receptors (PPARs) are ligand-activated nuclear hormone receptors that have pleiotropic immune modulating effects [21]–[23]. PPARα is expressed in liver, adipose tissue and placenta, where PPARα regulates lipid metabolism and anti-inflammatory pathways [24]–[26]. PPARα seems to decrease inflammation, mainly through direct interaction with NF-κB, causing inhibition of its signaling pathway or reducing the activated levels of NF-κB and subsequent inflammation [27], [28]. PPARγ is originally known to regulate adipocyte differentiation and lipid metabolism [29]. Recently, growing evidence points to PPARγ implication in the regulation of the immune response, particularly in inflammation control [30], [31]. The ligands of PPARγ inhibit proinflammatory cytokines synthesis in human gestational tissues, including the placenta [32]. Otherwise, a growing number of studies have highlighted the interaction of PPARγ and NF-κB [33], [34]. PPARγ could inhibit activation of NF-κB through several mechanisms and then repress NF-κB-mediated transcription of proinflammatory cytokines [35], [36]. Concluding the preliminary research PPARγ seemingly takes the most important role among PPARs in placenta differentiation and immunology, our current study therefore majorly to clarify the roles of PPARγ in the pathogenesis of ICP.

Placental cytokine production and action is developmentally regulated. Cytokines are the intercellular messengers of the immune system and intimately participate in many aspects of pregnancy, expressed by various cells and gestational tissues [37]. Their expression profile has been used to categorize immune responses and the functional status of the immune system. Although cytokines are secreted by a number of immune cell types, T cells have often been characterized as playing a key role in determining the nature of an immune response [38]. Virtually, all known cytokines have been found to be expressed in the human placenta although their temporal pattern of expression is still incompletely understood. Cytokines are produced by the Hofbauer cells considered as the placental resident macrophages, as well as by the syncytiotrophoblast and cytotrophoblast cells [39]. There is much evidence that the conjunction of placental and T cell derived cytokines is instrumental in the establishment of pregnancy. An increased cytokine release in maternal circulation is associated with gestational disorders such as diabetes and pre-eclampsia as a result of increased local production. Increased cytokine release may also contribute to the activation of specific inflammatory pathways. The role of placenta in the development of ICP has been noticed because the disappearance observed that the expression of tumor necrosis factor (TNF-α) was significantly increased in placenta of ICP women as compared with that in normally pregnant women, and the abnormality of pruritits and the normalization of liver tests would occur after the delivery of placenta [40]. It was of cell-mediated immunity and the imbalance of Th1/Th2 at the maternal–fetal interface in ICP was also observed.

Owing to the limitation of data available regarding immune pathology in ICP, the mechanism for the peripheral activation of immunity and the roles of immune pathology in the pathogenesis of ICP are obscure. And more, the fact that the expression and activation of NF-κB could be modulated by PPARγ through several mechanisms [39], and activation of NF-κB could modulated the expression of Th1/Th2 cytokines, but whether or not PPARγ could modulating the expression and activation of NF-κB has not been evidenced in ICP, thus, extensive investigation needs to be conducted to explore the mechanisms and the contributions of immune activation in ICP. Otherwise, activation of placental inflammatory pathways is also a feature of normal pregnancy and is increased in various placental pathologies, and PPARγ and NF-κB were also plays a key role in immune and inflammatory processes [41]–[43]. The aim of present paper is to measure the expression of PPARγ and NF-κB at mRNA and protein levels in placenta tissues of ICP women and cultured HTR-8/SVneo cell and the maternal serum levels of cytokines interleukin-4 (IL-4), IL-6, IL-12 and TNF-α, to further investigate the roles that PPARγ/NF-κB signaling pathway play in inflammation and immune responses in the possible mechanism of ICP and found new therapy targets for the treatment of ICP.

## Materials and Methods

### Reagents

The assay kits for IL-4, IL-6, IL-12 and TNF-α were purchased from Shanghai Xitang Biotechnology Co., Ltd. (Xitang Biotechnology, Shanghai, China). The assay kits for total cholesterol (TC), triglycerides (TG), high-density lipoprotein cholesterol (HDL-C), low density lipoprotein cholesterol (LDL-C), total bile acids (TBA), total bilirubin (TBIL), direct bilirubin (DBIL), cholyglycine (CG),alanine aminotransferase (ALT), aspartate aminotransferase (AST) and alkaline phosphatase (ALP) were purchased from Beijing Zhongshan Goldenbridge Biotechnology Co., Ltd. (Zhongshan Goldenbridge Biotechnology, Beijing, China) and Beijing Leadman Biotech Co., Ltd. (Leadman Biotech, Beijing, China). Trizol was a product of Invitrogen (Carlsbad, CA, USA). Taq DNA polymerase and reverse transcriptase were products of Thermo Scientific (Thermo Scientific, USA). The primers of PPARγ forward, CAGGAGCAGAGCAAAGAGGTA, reverse, CAAACTCAAACTTGGGCTCCA, NF-κB forward, 5′-GTGAGGATGGGATCTGCACT-3′, reverse, 5′-CCTTCTGCTTGCAAATAGGC-3′ and GAPDH forward, 5′-AGAAGGCTGGGGCTCATTTG-3′, reverse, 5′-GAAGACTGTGGATGGCCCCT-3′ were used for amplification by reverse transcription polymerase chain reaction (RT-PCR) were synthesized by Shanghai Sangon Gene Company (Sangon Gene Company, Shanghai, China). The commercial kits for protein extract were supplied by Nanjing KeyGen Biotech. Co. Ltd. (Keygen Biotech, Nanjing, China) and bicinchoninic acid (BCA) kit for protein concentration was supplied by Beyotime Institute of Biotechnology (Beyotime Institute of Biotechnology, Jiangsu, China). The following rabbit monoclonal antibody of PPARγ (CST-2443S) and rabbit polyclonal NF-κB (ab-7970) were purchased from Cell Signaling Technology, Inc (Cell Signaling Technology, Danvers, USA) and Abcam (HK) Ltd. (Abcam, Hongkong, China), respectively. The human placenta trophoblast cell line HTR-8/SVneo, an immortalized human trophoblast cell line established from first-trimester human cytotrophoblast cell and proved to be an important tool for the study of placental function, was a kind gift from Dr Charles H. Graham (Queen’s University, Ontario, Canada) [44]. Penicillin and streptomycin were purchased from (Sigma Chemical Co. St. Louis, MO, USA), and taurocholate acid (TCA) was purchased from (Steraloids, Newport, USA). All other reagents used in this study were of analytical grade.

### Patient Participation and Tissue Collection

Placental tissues were obtained from women who were hospitalized in department of gynecology and obstetrics of wuxi maternal and child health hospital, the affiliated hospital of Nanjing medical university. Written consent was received from women after full explanation of the purpose, nature and risk of all procedures used before surgery. The hospital ethic committee approved the consent forms and the protocols to utilize the tissue.

Forty consecutive pregnant women with ICP were enrolled in the study. The severity of ICP was based on the occurrence of fetal complications and the results of laboratory tests [45]. A control group comprised 42 consecutive healthy women with physiological pregnancies. All subjects were nulliparous Chinese women with singleton pregnancy. The women with ICP attending the department of obstetrics and gynecology between 1 January 2011 and 1 January 2012, the diagnosis of ICP was based on the following criteria: (1) the presence of pruritus, predominantly located on hands and feet, that resolved within hours or days after delivery; (2) the abnormalities in liver-function tests suggestive of ICP, such as serum levels of ALT and AST greater than 40 IU/L, respectively; (3) elevated levels of fasting total serum bile acid (SBA) >10 µmol/L; and (4) no skin lesions caused by systemic diseases that could lead to pruritus. All the patients were referred during pregnancy and were confirmed for the absence of infection by hepatitis viruses (HAV, HBV, and HCV). The exclusion criteria included autoimmune diseases, moderate to severe alcohol intake, biliary obstruction, and the use of drugs or alternative medicine therapy known to precipitate cholestasis. Information was provided by the same doctor to all patients on enrolment. Data on occurrence of cholelithiasis or other liver diseases were recorded according to information provided by the patients.

All the placental samples both from ICP and normal pregnancy were obtained immediately after caesarean sections. To avoid the further effect of labor on the expression profile, only placental samples that were obtained from the women who had not undergone labor were included. Four samples were taken at 0, 3, 6 and 9 o’clock of middle zone of each placenta after the deciduas and amniotic membranes were removed. We then dissected 1×1×1 cm sections of placental between basal and chorionic plates. Tissues was dissected and repeatedly rinsed until free from blood in chilled (4°C) PBS, dried with clean gauze, placed in dry vials, treated with diethylpyrocarbonate (DEPC) and snap-frozen in liquid nitrogen and stored at −80°C for RNA or protein extraction.

### Blood Samples Collection

The peripheral blood (5 ml) was collected from each participant in EDTA-containing tube. The blood sample was centrifuged within 2 h (4°C, 35,000 r/min, 10 min), and the supernatant was transferred to a sterile 1.5 ml centrifuge tube. All plasma samples were aliquoted and stored at −80°C until further use for IL-4, IL-6, IL-12, TNF-α, TBA, TBIL, DBIL, CG, ALT, AST, ALP, TC, TG, HDL-C and LDL-C measurement.

### HTR-8/SVneo Cell Culture Treatments

The human placenta trophoblast cell line HTR-8/SVneo was cultured in RPMI 1640 media supplemented with 10% charcoal-stripped fetal bovine serum (FBS), 100 U/ml penicillin and 100 µg/ml streptomycin under a humidified 5% CO_2_/95% air atmosphere at 37°C. After reaching 90% confluence, cells were trypsinized and passaged weekly. The cells were seeded in 6-well plates at a density of 2×10^5^ cells per well and treated after overnight culture. TCA is one of the main bile acids which elevated in patients with ICP. According to the method described in [46], the cells were divided into four treatment groups, and exposed to different concentrations of TCA; 10 µM, 50 µM and 100 µM TCA were used to represent the serum bile acid levels observed in normal pregnancy, mild and severe cholestasis, respectively. Cells treated with an equal volume of vehicle (distilled water) in media were used as negative control (0 µM). RNA and total protein lysates collected after 24 h for RT-PCR and Western blotting analyses.

### Serum Levels of TBA, TBIL, DBIL, CG, ALT, AST, ALP, TC, TG, HDL-C and LDL-C Measurement

Serum levels of TBA, TBIL, DBIL, CG, ALT, AST, ALP, TC, TG, HDL-C and LDL-C were determined by routine automated techniques.

### Inflammatory Cytokine Assay

The maternal serum levels of cytokines IL-4, IL-6, IL-12 and TNF-αwere measured by ELISA according to manufacturer’s instructions with modifications and the absorbance detected at 450 nm (corrected for background at 605 nm) on a VersaMax plate reader (Molecular Devices, CA, USA). Curve fitting and data extrapolation were performed using Soft Max Pro40 software (Molecular Devices, CA, USA).

### RNA Extraction and RT-PCR

According to the manufacturer’s instruction, total RNA was extracted from HTR-8/SVneo cells and placentas from patients with Trizol. Then total RNA (5 µg) was used for the RT reaction performed in 40 µL of the final volume at 42°C 60 min. The PCR conditions were as follows: 1 cycle of 94°C for 5 min, followed by 30 cycles of denaturation at 94°C for 45 s, annealing for 45 s, and extension at 72°C for 60 s. A final extension was 72°C for 10 min. The specific primers and annealing temperature were used for PCR amplification. The PCR products were separated on 1.5% agarose gel and quantitated by densitometry using an Image Master VDS system and the associated software (Pharmacia, USA). Data were expressed as ratio of the signals of interest band to those of GAPDH band. The latter acted as the internal control in the experiments.

### Protein Extraction and Western Blotting

Protein was extracted from HTR-8/SVneo cells and placentas from patients using the commercial kit in accordance with the manufacture’s instruction, and the protein concentration was determined by a bicinchoninic acid (BCA) kit. An aliquot of 60 µg protein from each sample was loaded onto 10% SDS-polyacrylamidegel and subjected to electrophoresis, and then transferred to nitrocellulose membranes. The membranes were blocked with 5% nonfat milk at room temperature for 2 h. Afterwards, the membranes were incubated with the primary antibodies for PPARγ (1∶1000 dilution), NF-κB (1∶1000 dilution), and β-actin (1∶2000 dilution) at 4°C overnight, then with fluorescent secondary antibody at room temperature for 1 h. The reaction products were quantified by densitometry using an Odyssey infrared imaging system and the Image J software. The ratio of the protein interested was subjected to β-actin, which acted as the internal control in the experiments.

### Immunohistochemistry

Placental tissue samples were fixed in buffered formalin for 24 h at 4°C, dehydrated in 70% ethanol, paraffin embedded, and sectioned. To improve the demonstration of antigens, we incubated slides with Protease K for 20 min to help uncover the hidden antigens. Deparaffinized placental sections were used for PPARγand NF-κB labeling. After the inhibition of endogenous peroxidases with 3% H_2_O_2_, the sections were incubated for 30 min at room temperature with 10% rabbit serum to block non-specific background signals. Serial sections were then incubated with specific primary antibodies against human PPARγ (1∶100) and NF-κB (1∶100) overnight at 4°C. After several washes in 50 Mm Tris-buffered saline and Tween 20, the slides were incubated with biotinylated goat anti-rabbit antibody (1∶2000) for 1 h at room temperature, sections were washed again with Tris-buffered saline and Tween 20 and secondary antibodies were detected by use of a DAB detection kit (Ventana; Roche, Indianapolis, IN). Negative controls were performed by omitting the primary antibody. The sections were examined by conventional light microscopy.

### Statistical Analysis

Unpaired *t*-test for comparisons between groups (Data are expressed as mean ± SD). Χ^2^-test was used to evaluate the comparisons of the rates. The statistical analysis was conducted using SPSS 13.0, p<0.05 was considered statistically significant.

## Results

### Clinical Characteristics of the Patients

Table 1 presents the clinical characteristics for the patients included in the evaluation of ICP women compared to control women. Maternal age, weight, height, gravidity, antenatal fetal heart rate, Apgar score ≤7 at 5 min and fetal demise were not significantly different between ICP and control women. Weeks gestational, birth weight, fetal complications, spontaneous preterm delivery, fetal distress and meconium-stained amniotic fluid in severe ICP group were significantly different as compared to that in control group. Accordingly, weeks gestational and birth weight in mild ICP group were also difference to that in control group (table 1).

**Table 1:** Clinical characteristics of ICP and control groups.

Perinatal characteristics	Control (n = 42)	Mild ICP (n = 20)	Severe ICP (n = 20)
Maternal Age (years)	26.5±4.3	26.7±4.5	27.9±5.3
Maternal weight (kg)	65.2±8.6	68.4±8.9	67.2±9.0
Maternal height (cm)	160.1±3.0	160.5±4.5	159.6±4.5
Gravidity (times)	1.4±0.8	1.5±0.8	1.6±0.7
Antenatal fetal heart rate	141.2±6.2	141.5±3.7	140.8±6.3
Weeks gestational (weeks)	38.7±1.2	36.9±2.7**	35.7±2.4**
Birth weight (kg)	3.38±0.33	3.0±0.50**	2.64±0.52**
Fetal complications (%)	1.2%	10%	20%*
Spontaneous preterm delivery (%)	2.4%	12.5%	47.5%**
Apgar score ≤7 at 5 min (%)	1.2%	5%	15%
Fetal distress (%)	2.4%	10%	30%**
Meconium-stained amniotic fluid (%)	4.9%	17.5%	32.5%*
Fetal demise (%)	N/A	N/A	5%

The data are expressed as the mean ± S.D., *p<0.05 vs. control group, **p<0.01 vs. control group. Χ^2^-test was used to evaluate the comparisons of the rates, *p<0.05 vs. control group, **p<0.01 vs. control group.

### Serum Biochemical Characteristics of the Patients

Table 2 presents the serum biochemical characteristics for the patients included in the evaluation of ICP women compared to control women. ALT, AST, ALP, CG, TBA, TBIL, DBIL, TC, TG, HDL-C and LDL-C in ICP group were significantly different as compared to that in control group (table 2).

**Table 2:** Serum biochemical characteristics of ICP and control groups.

Biochemical characteristic	Control (n = 42)	Mild ICP (n = 20)	Severe ICP (n = 20)
ALT (IU/L)	13.47±2.34	80.85±8.48**	107.47±9.96**
AST (IU/L)	15.97±2.72	62.32±6.92**	88.47±6.37**
ALP (IU/L)	158.53±17.85	200.57±21.04**	258.60±15.17**
CG (µg/dL)	2.97±0.92	31.15±4.02**	55.47±6.70**
TBA (µmol/L)	3.48±1.82	21.80±3.48**	67.88±13.39**
TBIL (µmol/L)	6.67±1.52	12.18±3.69**	17.64±5.63**
DBIL (µmol/L)	2.04±0.62	5.21±1.50**	10.15±2.80**
TC (mmol/L)	5.29±1.05	6.34±0.96**	7.37±1.43**
TG (mmol/L)	2.68±0.97	3.67±1.12**	4.34±1.02**
HDL-C (mmol/L)	2.34±1.12	1.62±0.92**	1.07±0.78**
LDL-C (mmol/L)	2.60±0.87	3.28±0.92**	4.27±1.06**

The data are expressed as the mean ± S.D., **p<0.01 vs. control group.

### Serum Cytokine Levels of the Patients

Assessment of the inflammatory cytokines were performed by measuring Th1 cytokines IL-12, Th2 cytokines IL-4 and another cytokines category, includes TNF-α and IL-6 and considered to exhibit proinflammatory function. ELISA analysis showed that the serum levels of TNF-α, IL-6 and IL-12 were significantly higher in ICP groups than in control group, and serum level of IL-4 in ICP groups was significantly lower as compared to that in control group (table 3).

**Table 3:** Serum biochemical characteristics of ICP and control groups.

Biochemical characteristic	Control (n = 42)	Mild ICP (n = 20)	Severe ICP (n = 20)
TNF-α (pg/mL)	67.85±17.92	310.24±54.37**	421.86±66.70**
IL-6 (pg/mL)	150.84±36.70	246.77±39.48**	387.62±65.68**
IL-12 (pg/mL)	24.13±4.51	58.39±18.97**	69.48±23.42**
IL-4 (pg/mL)	35.28±5.62	21.12±4.13**	11.63±2.74**

The data are expressed as the mean ± S.D., **p<0.01 vs. control group.

### Localization of PPARγ and NF-κB in Human Placenta

**Figure 1:**
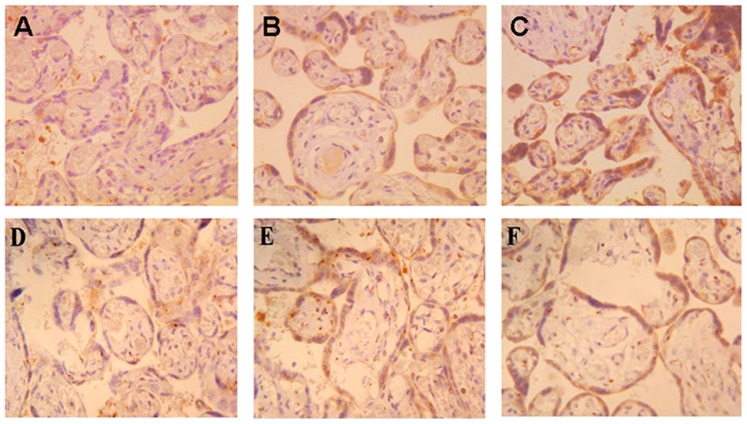
PPARγ and NF-κB staining were found in the membrane and cytoplasm of placental trophoblast cell. ×400 (A–C) PPARγ protein expressed in the placenta of control patients, mild ICP patients and severe ICP patients. (D–F) NF-κB protein expressed in the placenta of control patients mild ICP patients and severe ICP patients. PPARγ and NF-κB proteins expression were significantly different in control group and ICP groups.

PPARγ and NF-κB were identified in the placentas of both control and ICP patients using immunohistochemistry. Our results showed that PPARγ and NF-κB staining were found in the membrane and cytoplasm of placental trophoblast cell and PPARγ (A, B, C) and NF-κB (D, E, F) protein expressions in ICP patients were significantly higher than control patients (figure 1).

### Expression of PPARγ and NF-κB mRNA and Protein in Human Placenta

**Figure 2:**
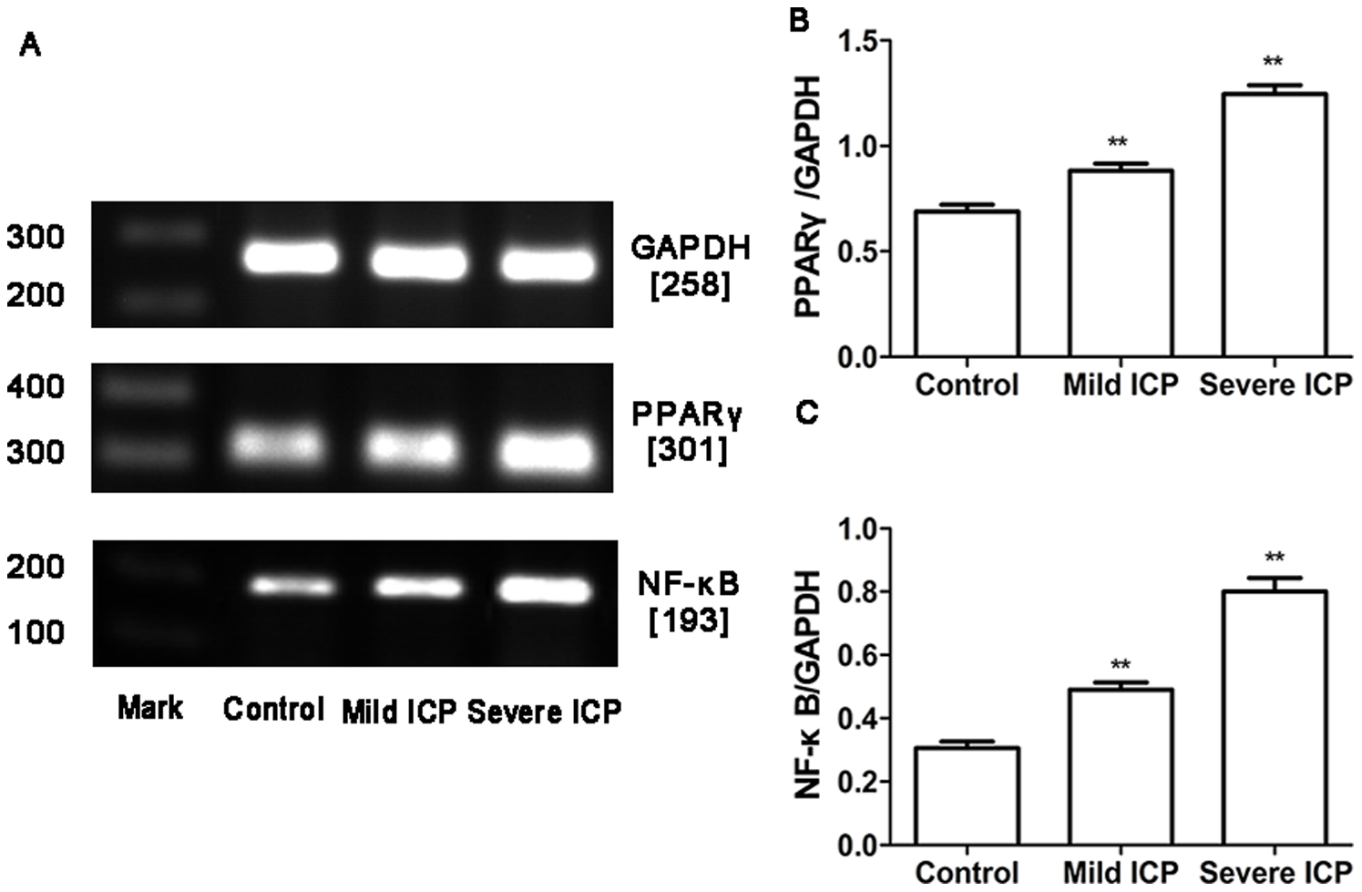
Expression of PPARγ and NF-κB mRNA in placentas from control group and ICP groups. (A) RT-PCR analysis of placental PPARγ and NF-κB mRNAexpression in control, mild ICP and sever ICP groups. (B) Graphical summary of data on the expression of PPARγ mRNA. (C) Graphical summary of data on the expression of NF-κB mRNA. The data are expressed as the mean ± S.D., **p<0.01 vs. control group.

**Figure 3:**
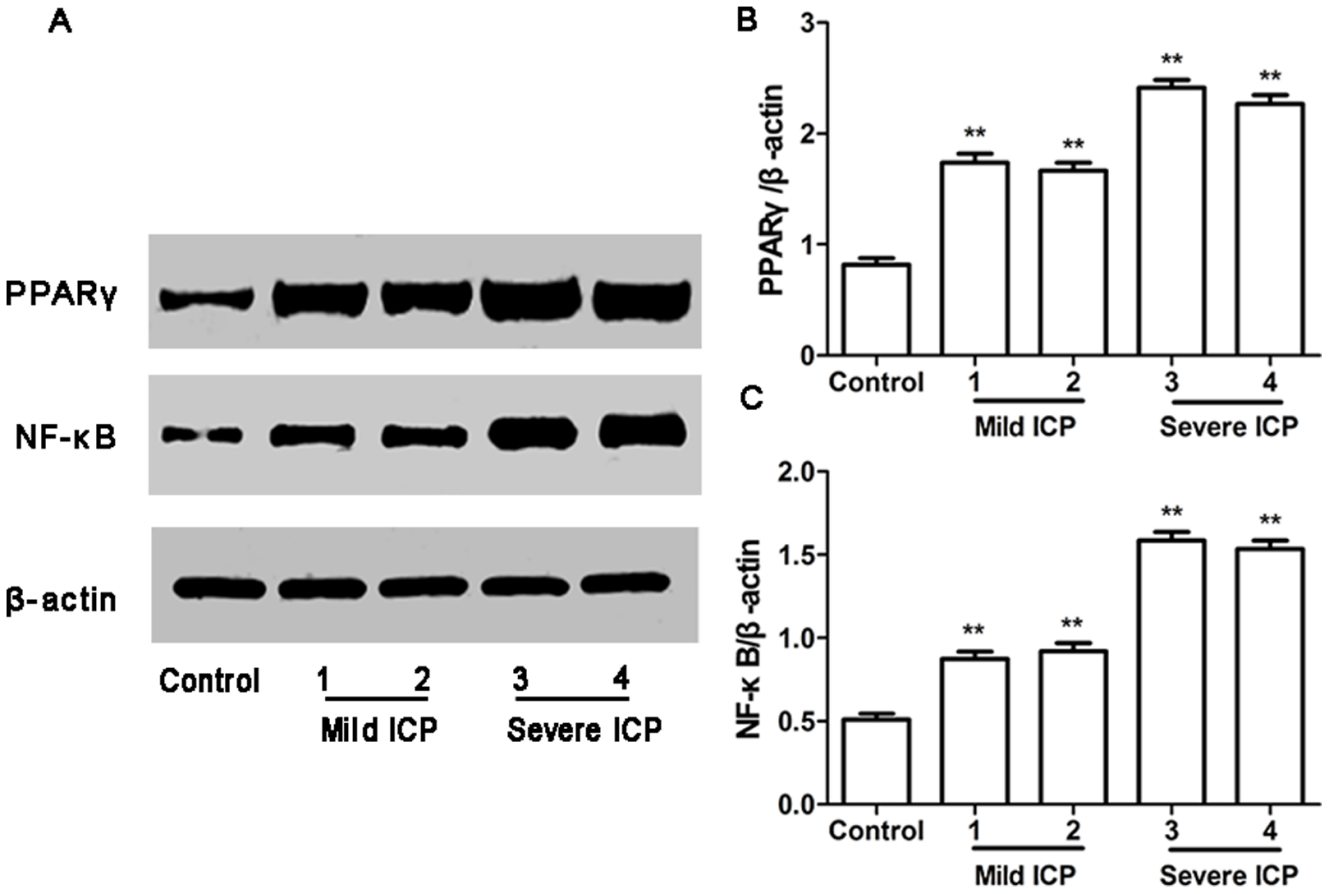
Expression of PPARγ and NF-κB protein in placentas from control group and ICP groups. (A) Western blotting analysis of placental PPARγ and NF-κB protein expression in control, mild ICP and sever ICP groups. (B) Graphical summary of data on the expression of PPARγ protein. (C) Graphical summary of data on the expression of NF-κB protein. The data are expressed as the mean ± S.D., **p<0.01 vs. control group.

RT-PCR and Western blotting were used to determine PPARγ and NF-κB mRNA and protein expression in the placentas obtained from control group and ICP groups. In parallel, β-actin was detected in all tested placental tissues with RT-PCR and Western blotting. PPARγ and NF-κB mRNA and protein expression showed significantly different in control group and ICP groups (figure 2, 3).

### Expression of PPARγ and NF-κB mRNA and Protein in HTR-8/SVneo Cell

**Figure 4:**
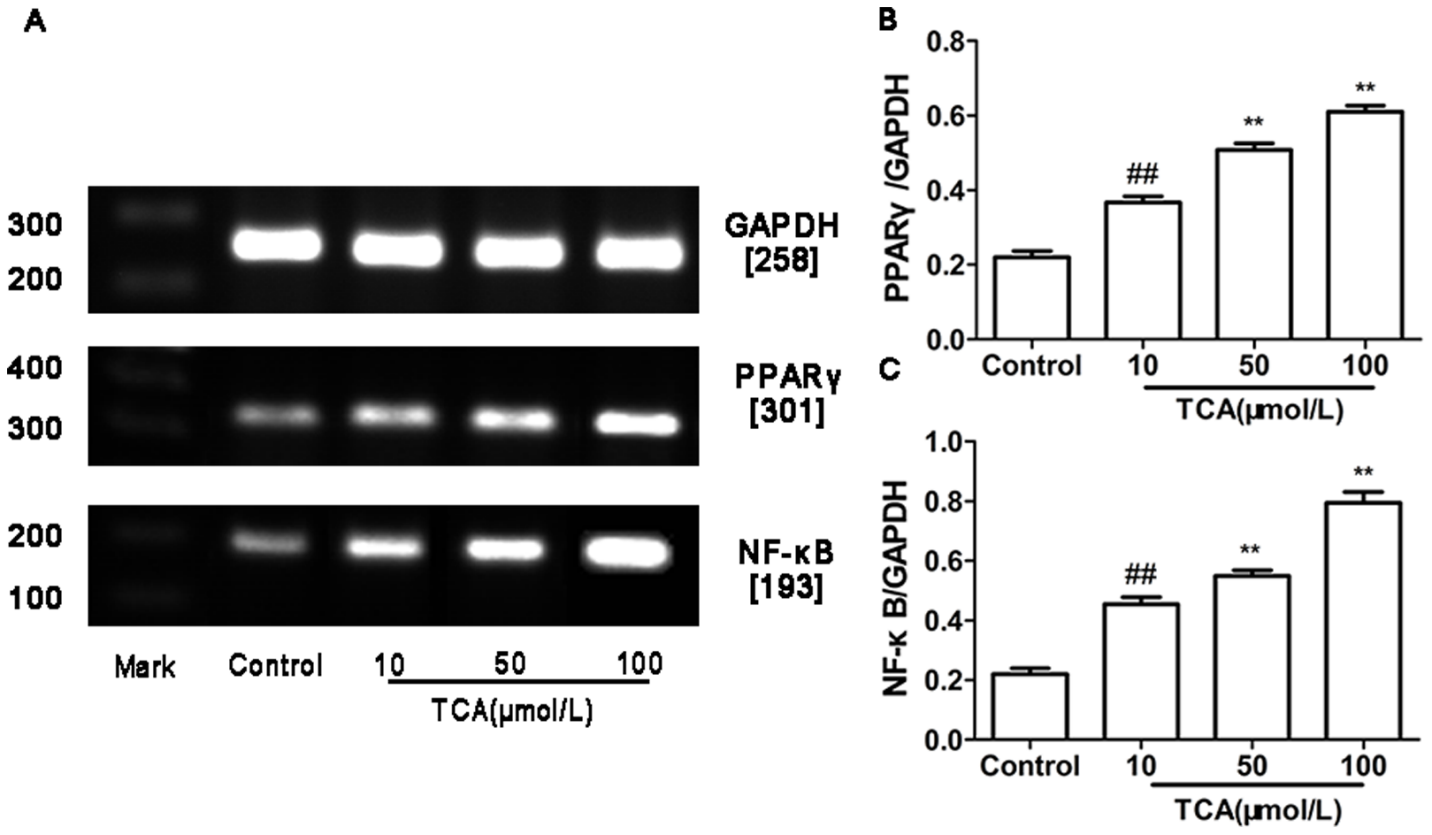
Expression of PPARγ andNF-κB mRNA in cultured HTR-8/SVneo cell. (A) RT-PCR analysis of placental PPARγ and NF-κB mRNA expression in control, mild ICP and sever ICP group. (B) Graphical summary of data on the expression of PPARγ mRNA. (C) Graphical summary of data on the expression of NF-κB mRNA. The data are expressed as the mean ± S.D., ^##^ *p*<0.01vs. control group, **p<0.01 vs. TCA 10 µM/L group.

**Figure 5:**
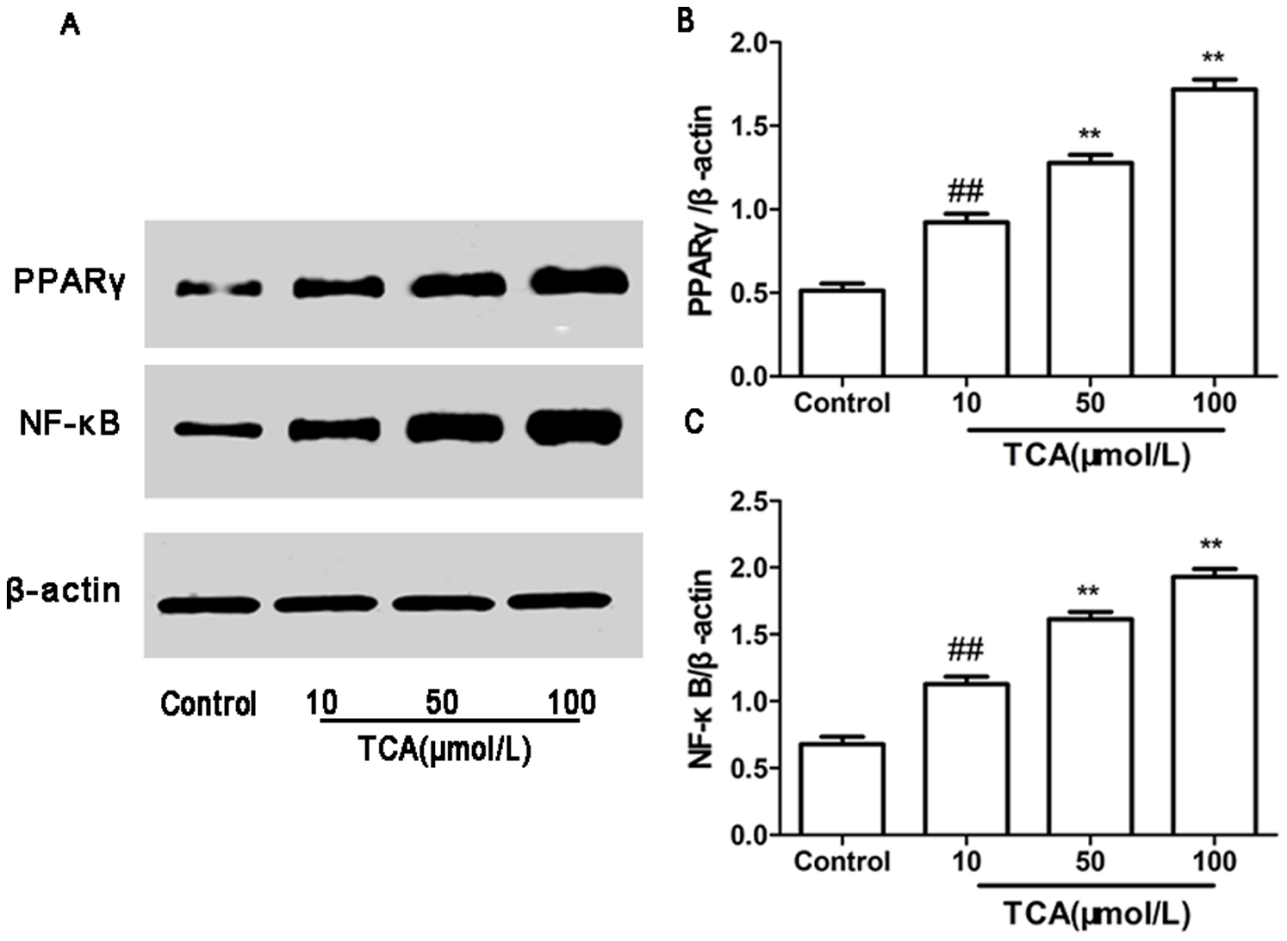
Expression of PPARγ and NF-κB protein in cultured HTR-8/SVneo cell. (A) Western blotting analysis of placental PPARγ and NF-κB protein expression in control, mild ICP and sever ICP group. (B) Graphical summary of data on the expression of PPARγ mRNA. (C) Graphical summary of data on the expression of NF-κB protein. The data are expressed as the mean ± S.D., ^##^ *p*<0.01vs. control group, **p<0.01 vs. TCA10 µM/L group.

RT-PCR and Western blotting were used to determine PPARγ and NF-κB mRNA and protein expression in cultured HTR-8/SVneo cell. In parallel, β-actin was detected in all tested placental tissues with RT-PCR and Western blotting. PPARγ and NF-κB mRNA and protein expression showed significantly different in control group and ICP groups (figure4, 5).

## Discussion

Our studies showed that PPARγ and NF-κB protein are predominantly expressed in the membrane and cytoplasm of placental trophoblast cell, and PPARγ and NF-κB mRNA and protein expression were significantly up-regulated in ICP placentas and TCA treated HTR-8/SVneo cells. The serum levels of cytokines IL-6, IL-12 and TNF-α were significantly increased in ICP patients, whereas the serum level of cytokine IL-4 was significantly decreased in ICP patients. The presented studies suggested that the higher expression of PPARγ and NF-κB and PPARγ up-regulated/dysregulation NF-κB expression and then induced abnormal serum levels of cytokines IL-4, IL-6, IL-12 and TNF-α, which might disturb inflammatory processes and the balance of immune reaction, placental bile acid and lipids transportation, resulted in fatal cholestasis and probably one of the mechanism of ICP.

Successful pregnancy has been associated with attenuated proinflammatory cytokine production in response to in vitro and in vivo immune challenges in human and animal studies. In recent years pregnancy has been found to be accompanied by a systemic inflammatory state as demonstrated by modest elevations in pro and anti-inflammatory cytokines in plasma of pregnant women [47]. During pregnancy, generation of distinct trophoblast cell types within the placenta is required to fulfill the complex biological processes of implantation, maternal fetal exchange and maternal tolerance of fetal parental antigens. What is more confounding is the fact that trophoblast cells as well appear to be the source of production of the proinflammatory cytokines.

Pregnancy is also a complex immunological state in which a bias towards Th2 protects the fetus and pregnancy is an anti-inflammatory condition, and that a shift in the type of cytokines produced would lead to pregnancy complications that are associated with additionally modified inflammatory responses [48], [49]. Th1 cytokines are potent inflammatory inducers, while those of Th2 type function as regulators to suppress inflammation and are known to down-regulate Th1 responses. Th1 cytokines closely linked to a positive pregnancy outcome [19]. IL-12 is a proinflammatory cytokine playing a key role in the polarization of the immune response toward Th1. IL-4 plays a central role in the orchestration of Type 2 immunity and considered to be anti-inflammatory cytokine [50]. In addition to an IL-4 positive feedback loop for Type 2 responses, IL-4 also acts as a negative regulator of Th1 inflammation [51].

Although IL-6 is produced by Th2 cells, it is considered to be a proinflammatory cytokine [52]. Proinflammatory cytokines such as IL-6 and TNF-α have been associated with pregnancy losses. IL-6 is a multifunction cytokine with a central role in immune regulation and inflammation [37], [53]. The function of TNF-α during pregnancy is multifaceted. It plays a pivotal role in inflammatory and immune responses. An appropriate amount of TNF-α is required in the regulation of the growth and differentiation of both the fetus and the placenta. However, excess amount of TNF-α triggers apoptotic intracellular signaling events which ultimately lead to embryonic death. It has been recently reported that an excessive amount of TNF-α may promote trophoblast cell apoptosis and directly damage the placenta [54]. Present studies also proved that the increase of TNF-α expression in placental trophoblasts was strongly associated with ICP pathology [55].

Inflammatory processes alter the balance of Th1 and Th2 cytokines causing a shift toward a Th1 predominance, which initiates and intensifies the cascade of inflammatory cytokine production involved in spontaneous abortion, preterm delivery, and other adverse pregnancy outcomes [56]. Some immune alterations also found in ICP, including imbalanced of CD4+/CD8+ lymphocyte and Th1/Th2 cytokines, increased levels of auto antibodies, impaired maternal-fetal mixed lymphocyte reaction, were reported similar to pregnancy failure and pregnant complications. ICP may be resulting from breach of the maternal-fetal immune tolerance during pregnancy. The appearance of antinuclear, antimitochondria, and antismooth muscle antibodies and increase in anticardiolipin antibody were also found in ICP. However, the understanding of immune alterations in ICP was much less.

The placenta provides critical transport functions between the maternal and fetal circulations during intrauterine development [57]. Placenta has been identified as a site of immune privilege, which is indirect contact with maternal cells in the uterine wall and with maternal blood. This tissue is the source of production of many immunomodulatory hormones and cytokines, and one prevalent hypothesis is that several of these factors released at the maternal-fetal interface or into the maternal blood stream contribute to the regulation of the local and systemic immune changes required for a successful pregnancy. By contrast, aberrant placental production of these factors has been shown to be associated with pregnancy-related disorders or even failed pregnancies [58]. Detailed molecular and cellular analyses of these immune changes are required for better understanding of placental physiology and pathology.

PPARγ is highly expressed in the human placenta, particularly in trophoblasts, and appears to have a pivotal role in trophoblast differentiation and placental development and function [59]–[63]. Besides the well-known role in lipid/glycidic homeostasis, PPARγ has also recently emerged as a key regulator of inflammatory and immune responses [64], [65]. The knockout of PPARγ gene in mouse highlighted its essential role in placental development. Investigations studying human placenta have shown that PPARγ is expressed in trophoblasts and circulating activators of PPARγ are increased in pregnancy and that activation of PPARγ can regulate trophoblast differentiation, invasion, and secretion of proinflammatory cytokines [32], [66]. Human clinical trials and animal studies also show that PPARγ agonists can have systemic anti-inflammatory effects. PPARγ ligands have been demonstrated to inhibit the secretion of IL6, IL8, and TNF-α in amnion and chorion, highlighting the role of PPARγ in the regulation of the inflammatory response in human gestational tissues and cells.

NF-κB plays a major role in the control of inflammation and immune responses, and controlling the expression of numerous genes involved in inflammatory and immune responses [67], [68]. NF-κB is involved in the regulation of proinflammatory cytokines in gestational tissues, including the placenta. Human clinical investigates shown that placenta NF-κB expression is higher in ICP women than the normal pregnant women, and placenta NF-κB expression is crossly relation with the severity of ICP [69]. Some recent studies demonstrated that NF-κB regulates the release of proinflammatory cytokines TNF-α, IL-6and IL-8 from intrauterine tissues [70], [71]. Additionally, a central role of NF-κB activation and ensuing trans-activation of promoters of inflammatory cytokines is cell degenerative processes.

Increasing attention is being paid to the cross-talk that occurs between nuclear receptors and signalling pathways regulating inflammation and immune responses. In this respect, cross-talk between PPARγ and NF-κB activity has been most intensely studied. The molecular mechanisms by which PPARγ exert anti-inflammatory effects is it physical interactions with NF-κB signalling either by inhibiting NF-κB DNA binding or by activating IκB kinase and then inhibit the expression of the majority of proinflammatory cytokines [72], [73]. Therefore, we studied PPARγ and NF-κB mRNA and protein expression in human placenta and cultured HTR-8/SVneo cells, and evaluated the human serum levels of cytokines IL-4, IL-6, IL-12 and TNF-α. We found that PPARγ and NF-κB protein are predominantly expressed in the membrane and cytoplasm of placental trophoblast cell, and PPARγ and NF-κB mRNA and protein expression are significantly up-regulated in ICP placentas and TCA treated HTR-8/SVneo cells. Assessment of the inflammatory cytokines were performed by measuring Th1 cytokines IL-12, Th2 cytokines IL-4 and another cytokines category, includes TNF-α and IL-6 and considered to exhibit proinflammatory function [38], [74]. Our studies showed that the serum level of IL-12 was significantly higher and IL-4 was significantly lower in ICP group than in control group, respectively, and proinflammatory cytokines TNF-α and IL-6 were significantly higher in ICP group as compared to that in control group. Our present results also demonstrated that the serum levels of ALT, AST and ALP in ICP group were significantly higher as compared to that in control group.

Together above findings support the view that ICP was associated with the suppression of Th2 immune responses that results in a Th1-biased status and that the increased levels proinflammatory cytokines in ICP were demonstrated the additionally inflammatory responses. Overall, the data presented herein suggested that higher expression of PPARγ and NF-κB mRNA and protein and PPARγ up-regulated/dysregulation NF-κB expression and then induced abnormal serum levels of cytokines IL-4, IL-6, IL-12, TNF-α and ALT, AST and ALP, which might disturb the balance of immune reaction, additionally inflammatory responses, placental bile acid and lipids transportation, resulted in fetal cholestasis, hepatic function injury and probably one of the mechanism of ICP.

Human pregnancy is a metabolic and immune challenge for the mother who has to accommodate in her womb a semi-allogenic fetus whose energy needs increase tremendously with gestation [58]. Though the etiology of ICP is not fully understood, it is likely associated with dyslipidemia, which may contribute to the pathogenesis of the disease [75]. PPARγ is essential for regulation of fat accumulation in trophoblasts and transport of fatty acids from the placenta to the fetus, and PPARγ also have crucial role in the regulation of bile acid metabolism. Human trophoblasts show increased uptake of lipids under PPARγ activation. It has been shown that null PPARγ placentas exhibit reduced lipid droplets in trophoblasts, whereas PPARγ and its heterodimeric nuclear receptor partner RXR enhance trophoblast fatty acid uptake and accumulation in vitro. Moreover, several proteins that modulate fatty acid transport and accumulation are expressed in placenta and are increased with the activation of PPARγ and/or RXR in primary human trophoblasts. As PPARγ mRNA and protein expression are significantly up-regulated in ICP placentas and TCA treated HTR-8/SVneo cells, and our results showed that the serum levels of CG, TBA, TBIL, DBIL, TC, TG, and LDL-C in ICP group were significantly higher as compared to that in control group, whereas serum level of HDL-C was significantly lower in ICP group than in control group. Then, the ICP women serum lipid and bile acid levels changed significantly, which might be due to the significantly up-regulated PPARγ mRNA and protein expression that regulate the higher levels of bile acid and accumulate fat for the pregnancy women increases energy needs.

ICP can lead to chronic placental insufficiency, affects the placenta morphologically by increase terminal villous and capillary surface area, and number of syncytial knots, resulting in fetal complications that include prematurity, meconium stained amniotic fluid, abnormal fetal heart rate, perinatal death, fetal distress, and stillbirth [76], [77]. Otherwise, the severity of ICP was based on the serum bile acid levels. With bile acid≥40 µmol/L, severe form of ICP patients suffered a significantly higher rate of fetal complications compared to women with normal bile acid levels and women with mild ICP [45]. So, we stratification of the ICP patients into two groups: “mild ICP” (bile acid levels of 10–39 µmol/L), and “severe ICP” (bile acid levels≥40 µmol/L). The fetal outcome data of our studies demonstrated that weeks gestational, birth weight, fetal complications, spontaneous preterm delivery, fetal distress and meconium-stained amniotic fluid in severe ICP group are significantly different as compared to that in control group, and weeks gestational and birth weight in mild ICP group was also differences to that in control group. Although the fetal demise has not significantly different between ICP groups and control group, but find one fetal death in severe ICP group.

In conclusion, this study provides novel information that the PPARγ-NF-κB-cytokine signaling pathway might play a significant role in the pathogenesis of ICP and provide a new approach to further investigate the etiology of ICP. Furthermore, our studies demonstrated that the fetal outcome data of ICP group were significantly different as compared to that in control group, taken together, these data demonstrate that PPARγ-NF-κB-cytokine signaling pathway might also play a key role in adverse pregnancy outcomes, which could be a potential targets for therapeutic intervention. The crosstalk between PPARγ and NF-κB at the placenta and HTR-8/SVneo cells level indicates an interaction between bile acid and lipids metabolism, hepatic function injury, fetal cholestasis and fetal complications and imbalance of immune reaction and additionally inflammatory responses induced diseases, such as ICP, and lead to new insights in pathogenesis and potentially also to novel treatment strategies for ICP. One of the limitations of the present study is that we did not assess placental expression of other PPAR isoforms and did not perform protein expression and DNA binding studies and the nuclear translocation of NF-κB, further more, suppression of the inflammatory and immune responses by PPARγ activation may also be achieved through induction of apoptosis of immune cells, which we might do in depth in future studies.
